# Pharmacodynamics and biodistribution of [195mPt]cisplatin(CISSPECT®) in head and neck squamous cell carcinoma

**DOI:** 10.1186/s13550-024-01082-w

**Published:** 2024-03-01

**Authors:** Reinout H. de Roest, Marijke Stigter van Walsum, Karlijn van der Schilden, Ruud H. Brakenhoff

**Affiliations:** 1 Otolaryngology/Head and Neck Surgery, Head and Neck Cancer Biology and Immunology laboratory, Amsterdam UMC Location Vrije Universiteit Amsterdam, De Boelelaan 1117, Amsterdam, The Netherlands; 2 Nuclear Research and Consultancy Group, Westerduinweg 3, 1755 LE Petten, The Netherlands; 3 Cancer Center Amsterdam, Imaging and Biomarkers, Amsterdam, The Netherlands

**Keywords:** Head and neck squamous cell carcinoma, Cisplatin, [195m]Platinum, Cisspect

## Abstract

**Background:**

Cisplatin- based chemoradiotherapy is a crucial pillar in the treatment of HNSCC. The use of cisplatin comes with high toxicity rates as 35% of patients cannot sustain the planned dose while response is unpredictable. Unfortunately, there are no clinically applicable biomarkers to predict response. Based on the association of response with the number of DNA adducts and the involved molecular pathway to resolve cisplatin-induced DNA crosslinks in HNSCC, [195mPt]cisplatin (CISSPECT®) might have potential to monitor drug uptake and retention before treatment, and predict cisplatin response. The aim of this study is to investigate this concept by analyzing uptake, retention and biodistribution of [195mPt]cisplatin between known cisplatin-sensitive (VU-SCC-1131) and –resistant (VU-SCC-OE) HNSCC cell lines in vitro and xenografted in mice in vivo.

**Results:**

By a variety of experiments in vitro, including cell cycle analyses, and in vivo, the sensitivity of cell line VU-SCC-1131 and resistance of cell line VU-SCC-OE for cisplatin was demonstrated. VU-SCC-OE was able to accumulate more [195mPt]cisplatin in the DNA, and showed an increased capability to repair [195mPt]cisplatin crosslinks compared to VU-SCC-1131. Notably, DNA binding of cisplatin increased even when cisplatin was removed from the medium, likely from intracellular sources. In vivo, [195mPt]cisplatin showed a rapid biodistribution to the large organs such as the liver, with no differences between intravenous and intraperitoneal administration. Most circulating [195mPt]cisplatin was cleared by renal filtration, and accumulation in kidney and liver remained high. Uptake in xenografts was rapid (blood:tumor ratio; 1:1) and highest after 1 h, while decreasing after 6 h in line with the concentration in the blood. Remarkably, there was no significant difference in uptake or retention between xenografts of the cisplatin-sensitive and -resistant cell line.

**Conclusion:**

VU-SCC-1131 with a known FA deficiency and VU-SCC-OE displayed a significant difference in sensitivity to and recovery from cisplatin treatment, due to S-phase problems in VU-SCC-1131 at low doses, in line with the genetic defect. Using Pt-195m radioactivity analysis, we demonstrated the limited capability of cisplatin crosslink repair in VU-SCC-1131. Unexpectedly, we were not able to translate these findings to a mouse model for sensitivity prediction based on the biodistribution in the tumor, most likely as other factors such as influx counterbalanced repair. These data do not support response prediction by [195mPt]cisplatin, and applications to predict the toxic side-effects of cisplatin and to tailor dosing schemes seem more feasible.

**Supplementary Information:**

The online version contains supplementary material available at 10.1186/s13550-024-01082-w.

## Background

Cisplatin was first discovered by Peyrone in 1844 as ‘Peyrone chloride’, and only in 1965 Rosenberg et al. found accidentally the growth inhibitory effect of this platinum compound on *E. coli* [1]. Soon the first experiments on anti-tumor effect of cisplatin were conducted, and promising results reported [2]. Cisplatin was approved by the FDA for treatment of testicular cancer and ovarian cancer in 1977 [3]. At the same period the first clinical trials in recurrent head and neck squamous cell carcinoma (HNSCC) were initiated with significant responses [4].

Since these first studies with cisplatin in HNSCC, this agent has become a crucial pillar in the primary, post-operative and palliative treatment settings of HNSCC. HNSCC often initially remains asymptomatic or only with mild symptoms resulting in a high proportion (~ 60%) of patients presenting with locally advanced disease. Advanced stage of disease is characterized by larger dimensions of the primary tumor often with invasion of anatomically neighboring structures and/or spread of the disease to the lymph nodes in the neck. In advanced stage oral cavity tumors, surgery has remained the mainstay of treatment, generally combined with post-operative radiotherapy or concomitant cisplatin-based chemoradiotherapy (CRT). For advanced HNSCC outside the oral cavity, surgery is often considered as too invasive with expectantly major consequences for functional outcome, and definitive concomitant CRT with high-dose cisplatin (100 mg/m2) every 3 weeks is the treatment of choice [5]. Furthermore, for recurrent or metastatic disease cisplatin is part of the standard of care (EXTREME regimen) combined with 5-FU and cetuximab. However, application of cisplatin comes with a price. It is a highly toxic treatment and 35% of patients do not sustain the treatment due cisplatin associated toxicity (e.g. neutropenia, nephro-, oto- and/or neurotoxicity) [6–8].

When cisplatin enters the cell, it undergoes the process of aquation in the low chloride intra-cellular environment which makes cisplatin more reactive to cellular targets. The aquated form of cisplatin covalently binds to its main target molecule, the DNA, leading to the formation of intra- and interstrand crosslinks (ICL) [9, 10]. This subsequently leads to cell cycle arrest and eventually tumor cell death. Sensitivity to cisplatin in HNSCC has been shown to be mainly determined by the level of cisplatin-DNA adducts, both in clinical and pre-clinical studies [11, 12]. This observation is in line with functional genomic studies that have shown the importance of the FA/BRCA pathway in the response to cisplatin, being the major pathway for sensing and repairing DNA-crosslinks [13]. Fanconi anemia (FA) is a recessive mainly autosomal genetic disorder characterized by congenital abnormalities, progressing bone marrow failure and cancer predisposition, most particularly acute myeloid leukemia and head and neck squamous cell carcinoma. There have been over 20 FA genes identified that work together in a complex to repair DNA crosslinks such as those caused by cisplatin.

Even though previous studies have led to better insight on the biological bases of the response to treatment with cisplatin, it has not resulted in clinically applicable imaging markers or biomarkers to predict response [14]. These are urgently awaited as cisplatin is toxic, and many patients may not benefit from this treatment, while they suffer from the adverse events such as leukopenia as well as nephro-, oto- and/or neurotoxicity [15–17]. Based on the assumption that the efficacy of cisplatin in HNSCC is mainly determined by the number of cisplatin-DNA adducts, we hypothesized that radiolabeled cisplatin is a potential agent to monitor uptake and retention of cisplatin in HNSCC and may serve as an indicator for treatment outcome by imaging.

Recently there has been more interest in radiolabeled cisplatin, which offers the opportunity to assess accurately the biodistribution of cisplatin [11, 18, 19]. Aalbersberg et al. described recently a reliable method to produce Pt-195 m with acceptable tracer activities and acquisition times, which enabled good image qualities in preclinical studies and accurate signal quantification [18]. Labeling cisplatin with Pt-195m is expected to provide comparable results in terms of imaging and quantification of biodistribution. The aim of this study is to get insight in the differences in biodistribution of [195mPt]cisplatin between known cisplatin-sensitive and -resistant HNSCC cell lines in vitro and in vivo.

## Methods

### Cell lines and culture conditions

We selected different HNSCC cell lines based on their sensitivity to cisplatin as described before [20]. The HNSCC cell lines VU-SCC-OE and VU-SCC-1131 were previously established at Amsterdam UMC location VUmc [21, 22]. The two cell lines are HPV-negative and near triploid [23]. VU-SCC-OE has recently been characterized as a typical HPV-negative HNSCC cell line with a classical copy number variation pattern and associated mutations in the known cancer driver genes (e.g. *TP53, PIK3CA*). The VU-SCC-1131 cell line is established from an FA patient with HNSCC. This cell line harbors a biallelic mutation in the *FANCC* gene [20, 21], the other morphological and genetic characteristics are very comparable to non-FA HNSCC cell lines with somatic mutations in the typical HNSCC driver genes and frequent copy number alterations [23]. However, the defect in the FA/BRCA DNA-crosslink repair pathway in VU-SCC-1131, causes a high sensitivity for cisplatin, while VU-SCC-OE has an intact FA pathway and is relatively cisplatin-resistant. Both cell lines were cultured in Dulbecco’s modified Eagle’s medium (DMEM), containing 5% fetal calf serum (FCS), 2mM l-glutamine. Cultures were maintained at 37 °C in a humidified atmosphere with 5% CO_2_.

### Determination of cisplatin sensitivity and recovery after exposure

Sensitivity of cell lines to cisplatin was determined by a serial dilution assay as described by Nagel et al. [20]. In short, cells were seeded in 96-wells plates at densities that allowed exponential growth during the time of the experiment. During the experiments, cells were maintained at 37 °C in a humidified atmosphere with 5% CO_2_. Cisplatin was added 24 h after plating in concentrations ranging from 666 µM–0.635 nM. Cell viability was assessed using a CellTiter-Blue assay (Promega, Leiden, The Netherlands) after 72 h continuous exposure (sensitivity assay) and after 4 h exposure combined with 72 h cisplatin-free medium (recovery assay). Dose-response curves were calculated using Graphpad Prism (version 9.0.0, GraphPad Software, Boston, Massachusetts USA).

### Cell cycle analysis

Cells were seeded in T25 flasks (Greiner) at 5 × 10^5^ cells per flask with conditions mentioned before. In total 24 hours after plating, cells were treated for 4 hours with 0.04 µM and 0.2 µM cisplatin. Subsequently, cells were rinsed and cultured with cisplatin-free DMEM, and harvested for further analysis at 0, 72 and 96 hours after medium replacement. Harvested cells were labeled with 10µM 5-ethynyl-2’-deoxyuridine (EdU) for ten minutes. After cell dissociation with trypsin and rinsing,,m,, with phosphate buffered saline (PBS), cells were fixed in 2% para-formaldehyde and resuspended in ethanol 70% end left overnight at -20 °C. Cells were permeabilized by incubation with the Triton X-100-based buffer (Thermo scientific, Waltham, United states) for 30 min at room temperature, cells were stained with mitosis marker Alexa Fluor® 647 anti-Histone H3 Phospho (Ser10) (Biolegend 650806, clone 11D8) and DAPI. BD LSR II Fortessa™ (BD Biosciences, Vianen, The Netherlands) and BD FACSDiva™ software (V8.0.1.1, BD Biosciences) were used for flow cytometry and data analysis.

### Production of Pt-195m radiolabeled cisplatin

Radioactive Pt-195m cisplatin was provided by NRG Advancing Nuclear Medicine (Petten, The Netherlands). Production of Pt-195m is described in the Additional file 1: Supplementary materials and methods, and synthesized according to a previously described protocol [24] In short, platinum-195m was produced by irradiation in the High Flux Reactor in separate batches (Pt-0009, Pt-0011 and Pt-0016). After irradiation, [195mPt]Cisplatin (CISSPECT®), or *cis*-[195mPt ][Pt(NH_3_)_2_Cl_2_], was synthesized according to published procedures [24] as a 1 mg/ml solution in 0.9% NaCl with a pH of 5 to 5.5. Part of the [195mPt]Cisplatin solution, containing 0.065–0.067 mg Pt, was analyzed for radioactivity and radionuclide purity (^197^Pt, ^191^Pt ^192^Ir, ^194^Ir, ^198^Au, ^199^Au) using a high purity Germanium detector coupled to a multi-channel analyzer system. The specific activity per mg Platinum at End of Irradiation (EoI) was 81MBq, 86 MBq and 131 MBq ^195m^Pt/mg Platinum for Pt-0009, Pt-0011 and Pt-0016 batches respectively. At the end of synthesis, 48 h after EoI, the Activity Reference Time (ART) was set, at ART the radionuclide purity increased to 93.9%, 93.6% and 99.1% for Pt-0009, Pt-0011 and Pt-00016 respectively due to decay of mainly ^197^Pt. [195mPt]cisplatin showed characteristic chemical properties as known for cisplatin, in line with the European Pharmacopoeia regulations for cisplatin (Additional file 1: Supplementary materials and methods).

### Intra-cellular retention of [195mPt]cisplatin and DNA repair analysis

Both cell lines were seeded at a density of 1 × 10^6^ cells per T25 flask (Greiner), and 2 days after seeding [195mPt]cisplatin (Pt-0011, Pt-0016) was added in three concentrations (5 µM, 20 µM and 75 µM). To assess uptake and retention of [195mPt]cisplatin we performed, the experiment under three conditions: (A) 4 h exposure to [195m]cisplatin and after which cells were harvested, (B) 4 h exposure to [195mPt]cisplatin after which the cisplatin-containing medium was removed, cells were rinsed with PBS and cisplatin-free DMEM was added and left for 24 h and (C) 24 h incubation with [195mPt]cisplatin after which cells were harvested. Cells were analyzed under the microscope for viability. For further analysis of all different fractions the cell suspension was centrifuged for 5 min at 500x*g*, the cell fraction was rinsed with PBS and collected. Cells were lysed and homogenized, and both RNA and DNA were isolated using the AllPrep DNA/RNA mini kit (Qiagen). The radioactivity of all fractions was determined using a gammacounter (LKB-Wallac, 1282 CompuGamma; Pharmacia, Woerden), and corrected for decay.

### In vivo experiments on cisplatin and radioactive cisplatin

Athymic nude-Foxn1^nu^ (Envigo) mice were subcutaneously injected with either the VU-SCC-OE or the VU-SCC-1131 HNSCC cell line on both flanks, with 2 × 10^6^ cells per site and randomized to either cisplatin or vehicle-control group. Tumor volume was measured with electronic calipers (V= (L x W x H) x 0.5 where V = volume, L = length, W = width and H = height). When tumors reached an average size of 100 mm^3^ (range 80-200mm^3^) drugs were administered. In vivo sensitivity was determined after intra-peritoneal injection of 5 mg/kg cisplatin (CDDP, Accord) at day 0 and 7 after randomization. Both conditions were tested in 3–5 mice per group with 1–2 tumors per mouse, injected sites were discarded from further analysis when there was no tumor growth at baseline. Tumor volume was measured every 2–3 days for 21 days, mice were euthanized when tumor volume exceeded 1000mm^3^ or invaded the skin.

For the analysis of the biodistribution of radioactive cisplatin, mice were randomized to the [195mPt]cisplatin (Pt-0009 and Pt-0011) group (intravenously or intra-peritoneal) or the control group (*n* = 3 per group). We injected 2.5 mg/kg of Pt-195 m solution, 8 days after production and delivery. Standards of radioactivity were taken from the injection solution. Mice were euthanized at 1, 2, 6 and 24 h after injection, organs were dissected and subsequently weighed and radioactivity was determined using a gammacounter (LKB-Wallac, 1282 CompuGamma; Pharmacia, Woerden). The rate of counts per minute (CPM) was normalized as the percentage of CPM of the injected fraction, and subsequently standardized to the organ weight in grams. All animal experiments were performed according to Dutch and EU legislations, and the protocol was approved by the Institutional Review Board on animal experimentation.

### Statistics

Baseline statistics were performed in Rstudio (R version 4.0.3 (2020-10-10)). Statistical analysis of comparative tumor growth repeated measures of xenografts were performed by fitting a linear mixed effects regression model, with time in days and each single mouse as random effects. We used the control mice to compute a growth speed using a linear regression model, with the regression equation per cell line we calculated an expected tumor volume per time point. The expected tumor volume was introduced in the linear mixed affects model as a random effect. Wilcoxon signed-rank test was used to compare tumor sizes at a predetermined timeframe.

## Results

### Sensitivity, recovery and cell cycle effects of cisplatin on VU-SCC-1131 and VU-SCC-OE

**Fig. 1:**
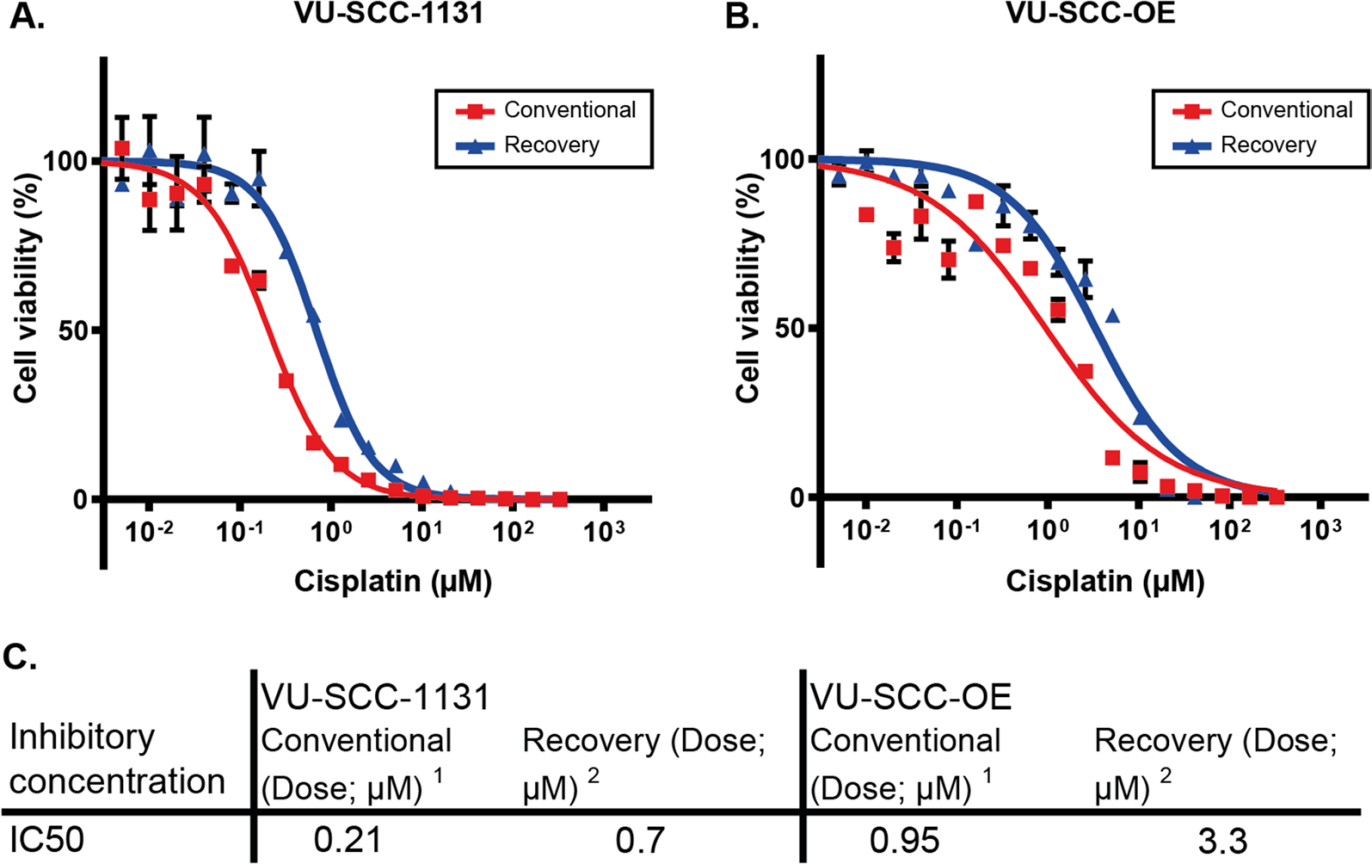
Dose-response curves of VU-SCC-1131 (**A**) and VU-SCC-OE (**B**). Table **C** shows IC50 values for both the conventional¹ and the recovery² experiment in cisplatin-free medium. ¹ (Conventional) Numbers represent percentage of cell death after a conventional dose-response experiment with 72 h continues cisplatin exposure. ² (Recovery) Numbers represent percentage of cell death after a dose-response experiment with 4 h cisplatin exposure and 72 h recovery in cisplatin-free medium

To assess in vitro drug response of the selected cell lines, we tested the sensitivity for cisplatin under continuous exposure, and the respective dose-response curves are shown in Fig. 1. As expected, the FA-deficient VU-SCC-1131 cell line was more sensitive for cisplatin than VU-SCC-OE, with IC50 values of 0.21 µM versus 0.95 µM respectively. These results were in line with previously reported IC50 values [20]. Effects after recovery were assessed by incubating cells for 4 h with cisplatin, replace the medium without cisplatin added and measure cell viability at 72 h. The half maximal inhibitory concentration shifted for both cell lines ~ 3 times to 0.70 µM for VU-SCC-1131 and 3.3 µM for VU-SCC-OE, respectively (Fig. 1). The difference of 5x in response remained constant.

**Fig. 2:**
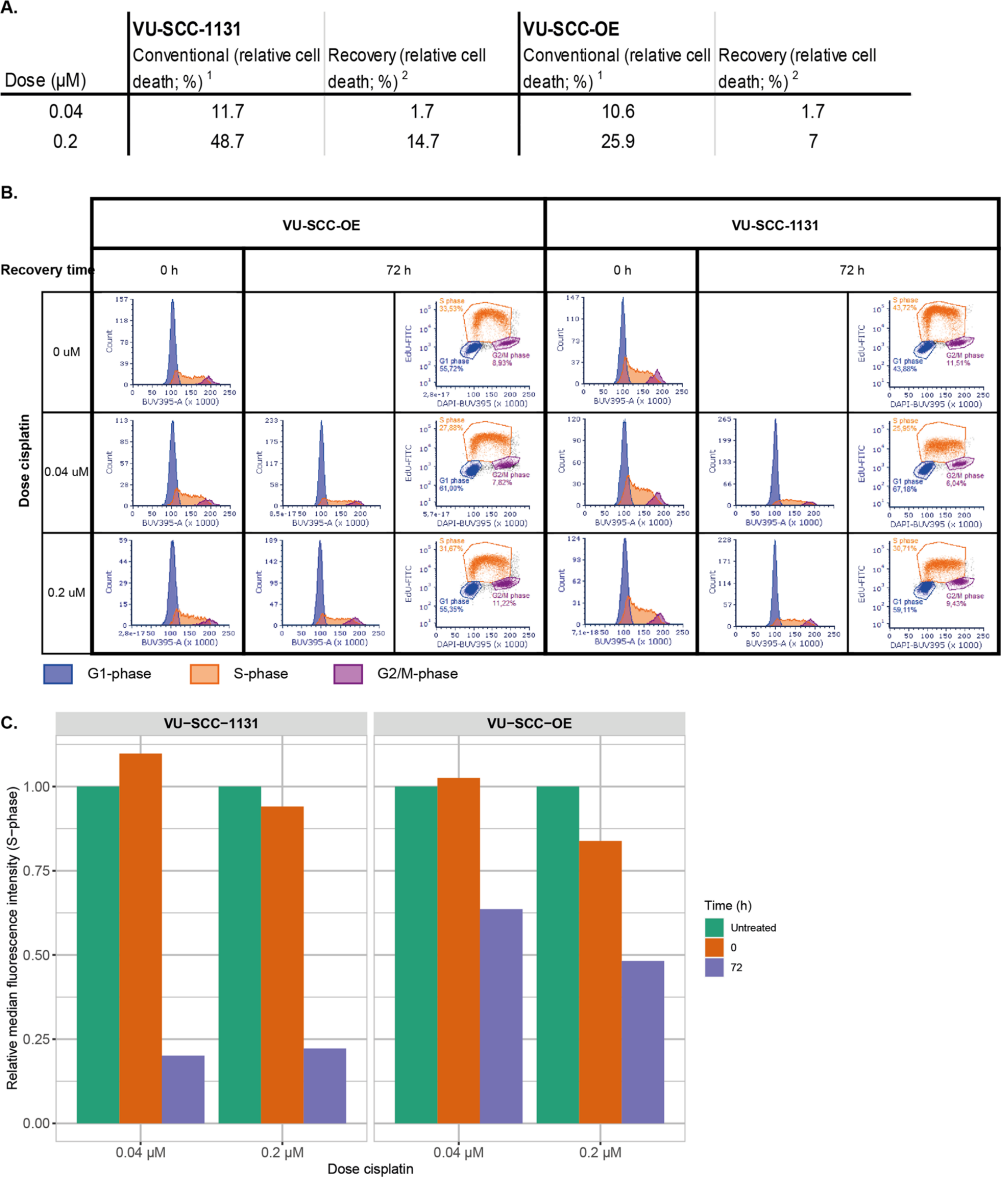
Panel **A** shows the percentage of cell death for both cell lines after treatment with either 0.04 µM or 0.2 µM cisplatin after either conventional dose-response analysis and after recovery, derived from Fig. 1A and B. Panel **B** shows the cell cycle distribution analysis after cisplatin exposure. Both cell lines were exposed for 4 h with low dose (0.04 µM) cisplatin which correlates with the IC1.7 value of both VU-SCC-1131 and VU-SCC-OE in the recovery experiments with cisplatin-free medium, and high dose (0.2 µM) which correlates with the IC14.7 of VU-SCC-1131 and the IC7.0 of VU-SCC-OE. Cell cycle distribution was assessed directly after exposure and after 72 and 96 h recovery in cisplatin-free medium allowing repair. **C**) analysis of median fluorescence intensity (MFI) of EdU in S-phase differs between cell lines. VU-SCC-1131 shows at low and high dose ~ 82% reduction of the MFI after 72 h recovery, while in VU-SCC-OE MFI drops much less after 72 h recovery

Next, we studied the cellular effects of cisplatin on the cell cycle using flow-cytometry analysis after EdU labeling in relation to dose, cell line and exposure time. EdU labeling allows high resolution DNA content analysis in combination with specific cell cycle markers. We treated cells for 4 h with cisplatin: a relatively low dose (0.04 µM) which correlates in our recovery experiment with the IC1.7 values of both VU-SCC-1131 and VU-SCC-OE, and a higher dose (0.2 µM) which correlates with the IC14.7 of VU-SCC-1131 and the IC7.0 of VU-SCC-OE (Fig. 2A). Independent of dose and cell line, analysis immediately after cisplatin exposure did not show any effects on the cell cycle compared to untreated samples as expected (Fig. 2B). However, VU-SCC-1131 accumulated in G1-phase after 72 and 96 h recovery time at both low and high doses (Additional file 3: Fig. S1A) The number of cells in mitosis did not change in VU-SCC-1131 when exposed to the low dose of cisplatin. However, when the high dose was applied, the fraction of cells in M-phase increased with longer recovery time (Additional file 3: Fig. S1B). VU-SCC-OE did not show major alterations in the distribution in either G1, S or G2/M-phase after exposure to both concentrations. Nonetheless, 2–3 times higher numbers of cells stayed in mitosis during recovery phase, dependent on the dose (Additional file 3: Fig. S1B). Although the cell numbers did not change dramatically, the EdU incorporation during S-phase was severely hampered, particularly in VU-SCC-1131 cells (Fig. 2B). The median fluorescence intensity (MFI) decreased substantially more in the cisplatin-sensitive VU-SCC-1131 cell line compared to the resistant VU-SCC-OE cell line after 72 h recovery time (Fig. 2C). Likely as result of the compromised DNA repair mechanism in VU-SCC-1131, the number of cross-links remained present, the replication forks collapsed and DNA synthesis was inhibited. These observations in cell cycle dynamics associate well with the sensitivity to cisplatin.

**Fig. 3:**
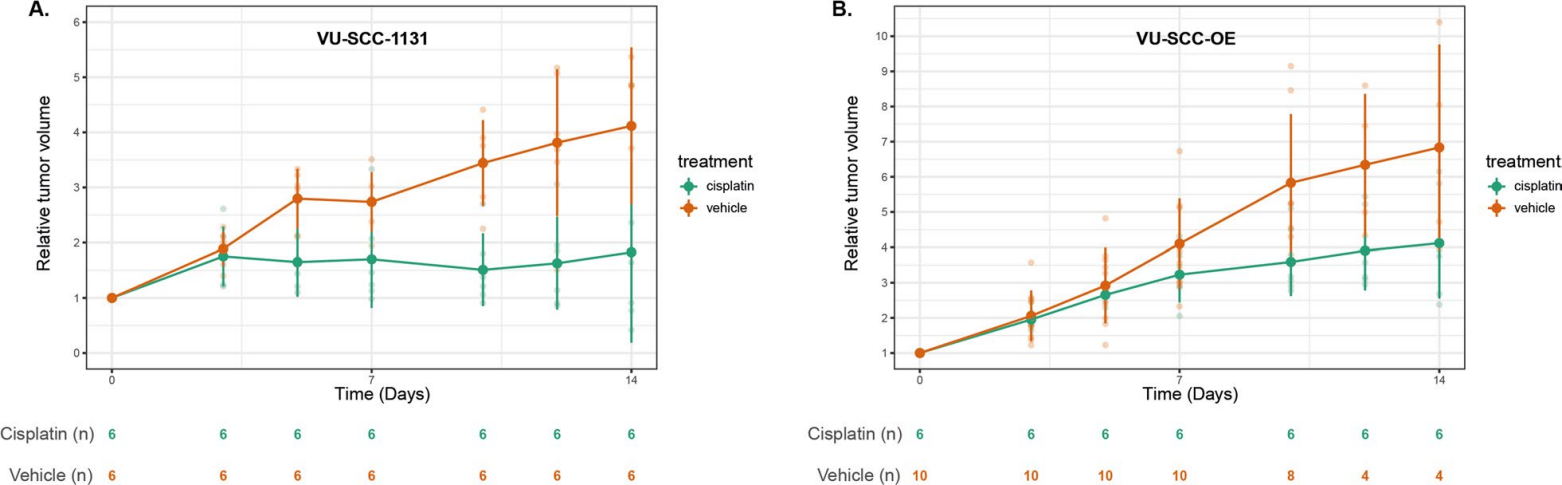
Shows the in vivo response of xenografts of both cell lines on cisplatin and vehicle (control). In total 6 mice were injected on both flanks with 2 × 10^6^ VU-SCC-1131 (**A**) cells per site and randomized to either cisplatin or control group, all tumors grew out sufficiently for further analysis. For VU-SCC-OE (**B**) 10 mice were injected with cells on both flanks and randomized to either a cisplatin or control group. Mice were treated intra-peritoneal with cisplatin 0.5 mg/kg or the control-vehicle (solvent) at days 0 and 7

Next we tested whether the differences in sensitivity in vitro in the cell lines, are also observed in vivo. In total 10 mice were injected on both flanks with 2 × 10^6^ VU-SCC-OE cells per site and randomized to either cisplatin or control group. In the control group all 10 sites with injected cells developed into tumors, while in the treatment group 6 of 10 sites with injected cells developed into tumors. For VU-SCC-1131 6 mice were injected with cells in both flanks, the mice were randomized over treatment and control groups, and all tumors grew out for further analysis. Mice were treated intra-peritoneal with cisplatin 0.5 mg/kg or the control-vehicle at day 0 and 7. Growth curves are depicted in Fig. 3. Although the take rates were more or less comparable, the two tumor models displayed a different growth rate. The VU-SCC-1131 doubled every 10 days while the VU-SCC-OE model doubled every four days. We observed that VU-SCC-1131 xenografts were more sensitive to cisplatin compared to VU-SCC-OE xenografts, resulting in larger growth delay factor in VU-SCC-1131 (1.61 and 2.28 at day 7 and day 10 respectively) versus VU-SCC-OE (1.27 and 1.63). However, to analyze that this was a significant difference, we fitted a linear mixed effects model for both cell lines with relative tumor volume as the outcome variable and the cell line (cisplatin vs. vehicle) as the fixed effect, we included time in days, each individual mouse and growth rate as random effect. This allows to analyze cisplatin efficacy in these two models while correcting for the difference in basic growth rate. The overall model predicting relative tumor volume explained 74.1% of the variance. The variance explained by the fixed effects was 16.3%. The model’s intercept is at 2.13 (SE = 0.58, 95% CI [0.98–3.29]). The reduction in tumor growth by cisplatin treatment was significantly different between xenografts of both cell lines, VU-SCC-1131 showed significant increased growth delay when compared to VU-SCC-OE (beta = 1.73, SE = 0.64, 95% CI [0.46–3.00], *p* < .01).

### Uptake and retention of [195mPt]cisplatin

The experiments described above, indicate that there is a significant difference in cisplatin sensitivity between the two cell lines, and the cell cycle analyses indicate that the data are well explained by the known genetic DNA crosslink repair defect. Next we analyzed using these models, whether this difference is reflected by [195mPt]cisplatin DNA adducts in vitro as well. In vitro uptake and retention was evaluated by direct measurement of [195mPt]cisplatin bound to nuclear DNA.

**Fig. 4:**
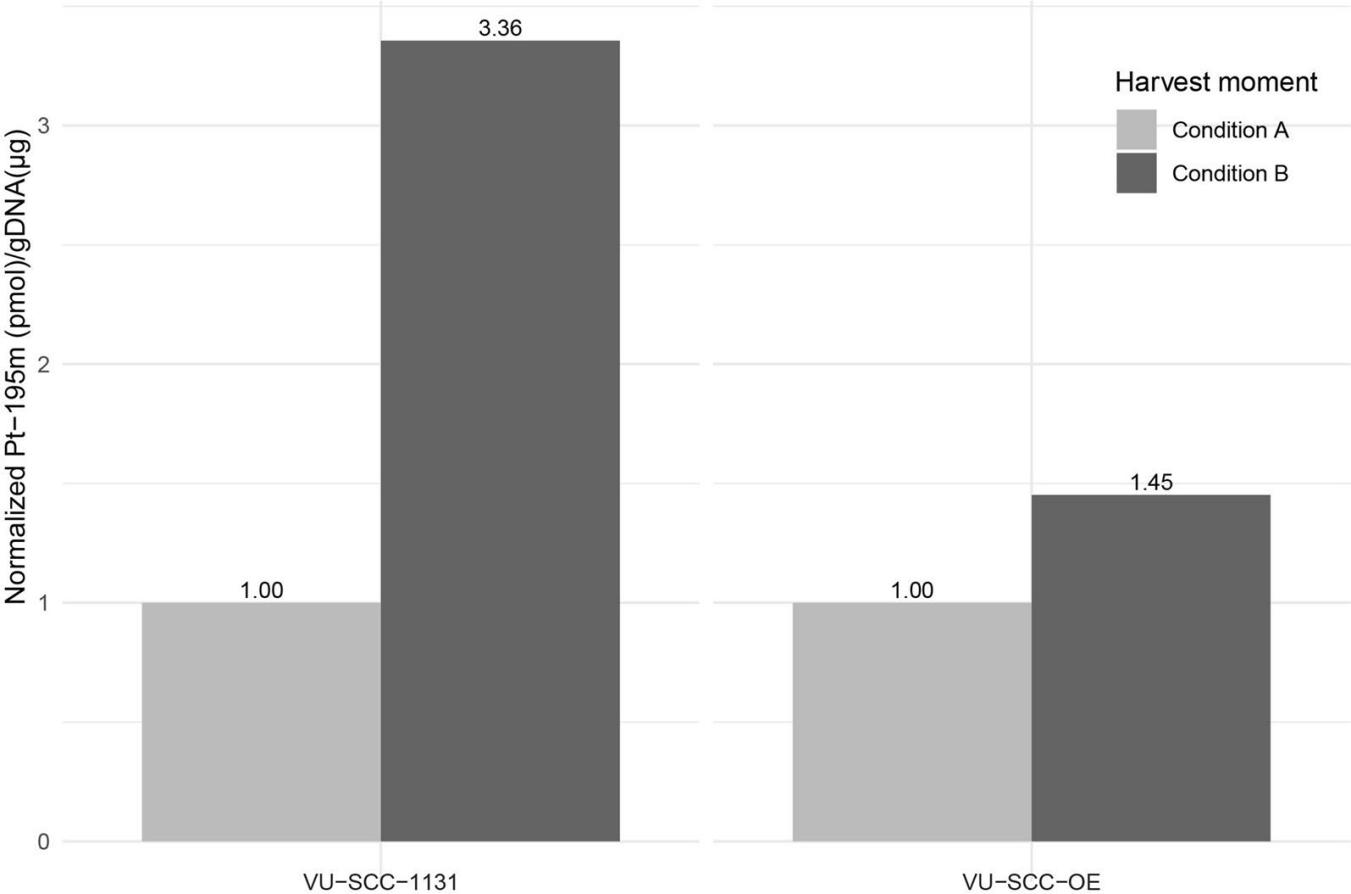
Shows the retention of [195mPt]cisplatin in the genomic DNA after 4 h exposure of 75 µM (**Condition A**) and after 4 h exposure and 24 h recovery time with refreshed drug-free medium. (**Condition B**)

The amount of [195mPt]cisplatin DNA adducts was measured after 4 and 24 h incubation. The amount of DNA-bound cisplatin has an incremental increase with both the concentration and the incubation time as expected (Additional file 3: Fig. S2). Against our expectations, both at 4 and 24 h incubation the insensitive cell line VU-SCC-OE accumulated a higher amount of cisplatin in the DNA compared to VU-SCC-1131, which may be the net result of a higher influx or lower efflux. This initial difference in accumulation needs to be taken into consideration when DNA repair is quantified after cisplatin removal, and to compensate for this effect we calculated the ratio with and without allowing repair (Fig. 4). To obtain enough radioactivity counts after 24 h repair and the associated decay of the radionuclide, we had to increase the dose to 75 µM, which is relatively high. As the combined incubation periods are short, we concluded that this was acceptable. Remarkably, when the medium was replaced without cisplatin, the accumulation of [195mPt]cisplatin in the DNA further increased in both cell lines. We explained this counterintuitive observation by assuming that there are intracellular cisplatin sources of stored or protein bound pools that apparently also accumulated in the DNA. In VU-SCC-OE the additional accumulation was counterbalanced by active crosslink repair, and the ratio was a mere 1.45. However the accumulation in VU-SCC-1131 increased tremendously with a ratio of 3.4. Together this suggests that the insensitive cell line VU-SCC-OE cleared DNA adducts from the DNA while the sensitive cell lines VU-SCC1131 did not, or considerably less. The higher sensitivity for cisplatin, the increased effect on S-phase arrest of cell line VU-SCC-1131, and the decreased removal of cisplatin adducts from the DNA, are all in line with and can be explained by the FA-deficient phenotype. As the two different cell lines can be grown as xenograft in immune deficient mice and display a comparable difference in cisplatin sensitivity, we tested radiolabeled cisplatin accumulation in a biodistribution experiment to test the possibility for differential uptake and possibly imaging.

### Biodistribution of Pt-195m labeled cisplatin in mice

**Fig. 5:**
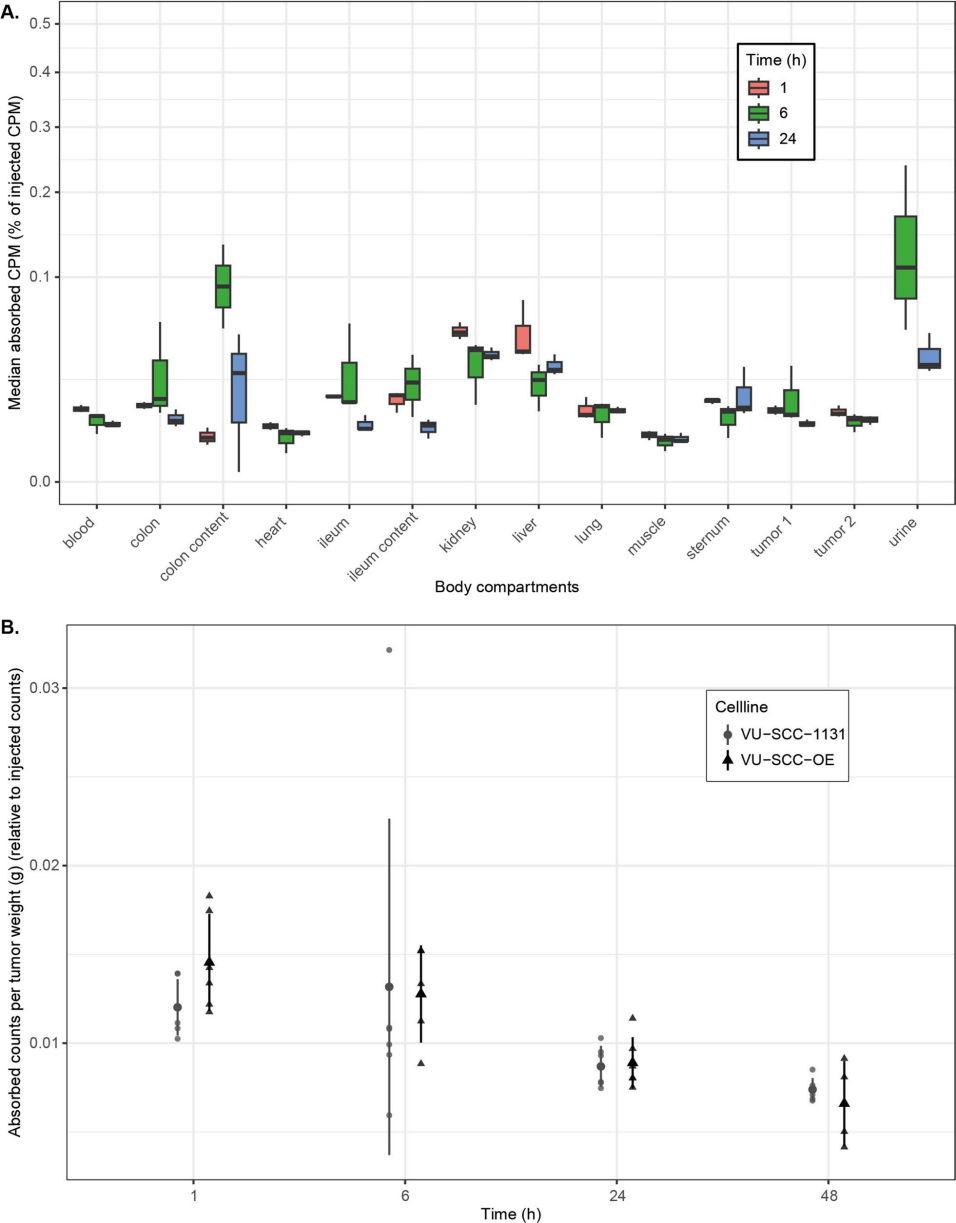
**A** Biodistribution of [195mPt]cisplatin in VU-SCC-1131 xenograft–bearing adult female nude mice (*n* = 3/time point) at 1, 6 and 24 h after intravenous injection. **B** Uptake over time in VU-SCC-1131 (cisplatin sensitive) and VU-SCC-OE (cisplatin resistant) xenografts. Radiotracer uptake is depicted as % injected dose/g tissue, and was determined by γ-counting and compensation for decay

Figure 5A shows the biodistribution of [195mPt]cisplatin in xenograft bearing mice after 1, 6 and 24 h. The administered [195mPt]cisplatin is rapidly, within the first hour, distributed to each organ. After the first hour, biodistribution was relatively consistent between 6 and 24 h. Most of the circulating [195mPt]cisplatin is cleared through glomular filtration, resulting in high accumulation of [195mPt]cisplatin in the urine fraction, not uncommon for these small molecules. Accumulation of [195mPt]cisplatin remained high in the main clearing organs over time, with median renal/blood and liver/blood AUC ratios of 4.31 (2.82–4.41) and 2.46 (2.33–3.04) respectively. There were no significant differences in the distribution to the large organs after intravenous (IV) or intra-peritoneal (IP) administration of the [195mPt]cisplatin solution (Additional file 2: Tables 1 and Additional file 3: Fig. S3) Likewise, the amount of uptake and retention in the xenografts was comparable and not significantly different between IV and IP drug delivery (Additional file 3: Fig. S4).

### Uptake and retention of Pt-195 m labeled cisplatin in xenografts

Figure 5B shows the uptake and retention of Pt-195 m labeled cisplatin in xenografts of the injected sensitive and non-sensitive cell lines. In the xenografts of both cell lines the uptake takes place within the first hour, with a maximum uptake at the 1 h time point of 0.012 (0.010–0.014) for VU-SCC-1131 and 0.014 (0.012–0.018) for VU-SCC-OE median absorbed counts (as percentage of injected counts). Retention of [195mPt]cisplatin decreased with time, with lowest amounts after 48 h in both xenograft mouse models (VU-SCC-1131 vs. VU-SCC-OE; $\stackrel{\sim}{\mathcal{x}}$ = 0.007 (0.007–0.009) vs. $\stackrel{\sim}{\mathcal{x}}$ = 0.007 (0.004–0.009)). A Wilcoxon signed-rank test indicated that the [195mPt]cisplatin uptake in the sensitive cell line (VU-SCC-1131) was not statistically significantly higher than in the resistant cell line (VU-SCC-OE) Z = 7, *p* = .09 (Fig. 1B) at the earliest time point, and remained comparable at subsequent time points (6 h: Z = 9, *p* = .33; 24 h: Z = 15, *p* = .70; 48 h: Z = 13, *p* = .91). Also, the course of the uptake and retention measured by the AUC of the median percentage of absorbed counts-time curve was comparable between both xenograft mouse models (VU-SCC-1131: $\stackrel{\sim}{\mathcal{x}}$ = 0.41 and VU-SCC-OE: $\stackrel{\sim}{\mathcal{x}}$ = 0.45), and the difference was not statistically different (Z = 19, *p* = .94).

## Discussion

Estimation of the in vivo distribution of platinum-based compounds is challenging, in particular in relation to response prediction. A variety of methods have been used to quantify the levels in the past [25], such as flameless atomic absorption spectrometry (FAAS) [26], gas chromatography-mass spectrometry [27] and liquid chromatography coupled to tandem mass spectrometry (LC/MS/MS) [28]. Although these methods have high sensitivities for detecting small amounts of platinum, they demand biopsies, are often time consuming, complex and high in costs and on the edge of detection limits. [Pt-195 m]-labeled cisplatin would allow reliable, direct, real-time and non-invasive analyses of cisplatin biodistribution, with comparable results to FAAS [18]. The production in a high-flux reactor and the rapid decay resulted in a limited number of biological replicates in the in vitro and in vivo experiments in our studies. This limitation should be taken into account while interpreting the results.

The FA-deficient cell line VU-SCC-1131 and cell line VU-SCC-OE displayed a significant difference in cisplatin sensitivity in vitro which was substantiated by an increased S-phase effect on the cell cycle, and an increase of radiolabeled cisplatin adducts after cisplatin-free recovery in cell line VU-SCC-1131. In vivo the difference in sensitivity was observed as well but the interpretation was somewhat hampered by the clearly lower doubling time of VU-SCC-1131 in vivo, which required more refined analyses. This difference in growth rate was not observed at all in vitro, and might be caused by the FA defect in VU-SCC-1131 hampering growth in mice. Analyses of the biodistribution of Pt-195 m radiolabeled cisplatin in mice showed rapid distribution to organs, high accumulation in kidneys and liver and high renal clearance. These results are in line with previous studies using [Pt-195 m]-radiolabeled cisplatin [18, 29–31], but also with clinical studies on pharmacodynamics of cisplatin in human patients [19, 32, 33]. Accumulation in kidneys was higher than in the liver tissue 24 h after administration of [195mPt]cisplatin, while in previous studies in mice it was shown that on the long-term the liver tissue will have a higher retention compared to the kidneys [34]. Nephrotoxicity is a major clinical problem in the clinic, with failure of treatment or exclusion of cisplatin-based treatment as result. Although Pt-195m radiolabeled cisplatin is suitable for imaging studies [18, 19], the high uptake and retention in liver and kidneys results in high signals in the abdominal region, which hampers imaging in the supra-diafragmal region [19]. Also in our study, scintigraphy of [195mPt]cisplatin was compromised by this pharmacokinetic property, as the xenografts were overshadowed by the high uptake in the liver and kidneys (data not shown).

Martens- de Kemp et al. showed that the amount of DNA-bound cisplatin after 4 h exposure was inversely correlated with the IC50 value of HNSCC cell lines [11]. These experiments were performed with 75 µM cisplatin, which is relatively toxic dose, but was required to obtain enough counts after DNA isolation. Remarkably, we noted that there is higher uptake in the non-sensitive cell line VU-SCC-OE likely by an increased influx or decreased efflux. When correcting for this effect, FA-deficient cell line VU-SCC-1131 demonstrated a much higher level of DNA adducts after recovery in cisplatin-free medium. As mentioned, in HNSCC the FA/BRCA pathway has been identified as the major pathway for repairing DNA-crosslinks [13]. The impairment of the FA/BRCA pathway in VU-SCC-1131 results in an impaired repair of the cross-links formed by cisplatin, and consequently increased retention of [195mPt]cisplatin in the DNA.

The intra-tumor uptake of [195mPt]cisplatin in mouse xenografts is rapid, with the maximum one hour after administration. Unexpectedly and disappointingly, the uptake in the xenografts at any time point was not significantly different between the sensitive and the resistant cell line. We noted a consistent activity in the blood with slow clearance, which might have skewed any differences within the tumor. Cisplatin is a highly reactive compound and might bind to cells and proteins in the blood. In addition we noted that insensitive cell line VU-SCC-OE showed an increased influx or decreased efflux in relation to cell line VU-SCC-1131, which might have counterbalanced any difference in tumor uptake caused by the FA defect to remove DNA adducts. This observation indicates that imaging differences as readout for predicting response, is disappointingly not a promising approach.

## Conclusions

In conclusion, although our in vitro and in vivo experiments show differences in retention of cisplatin between a sensitive and a non-sensitive HNSCC cell line, the translation to a theranostic tool of [195mPt]cisplatin was not successful, as differences between uptake and/or retention in xenografts were not observed. Our in vitro experiments showed that the observed differences between our cell lines could be attributed to the repair of DNA adducts in S-phase. However, we also noted differences in influx and efflux between cell lines and this phenomenon combined with the consistent activity in the blood might have overshadowed any difference in biodistribution between the sensitive and resistant xenografted cell lines. Hence, predicting tumor response using radiolabeled cisplatin in head and neck cancer patients seems not a rewarding approach. Alternatively, [195mPt]cisplatin could be used to get insights in the toxic side-effects of cisplatin. In addition, radiolabeled cisplatin might have an additional therapeutic effect, but also an increased toxicity when administered in high doses. These topics should be the focus of future studies.

### Supplementary Information

**Additional file 1**. Supplementary materials and methods describing the production of Pt-195m radiolabeled cisplatin.

**Additional file 2**. Supplementary figures.

**Additional file 3**. Supplementary table describing biodistribution of [195mPt] cisplatin in VU-SCC-1131 xenograft–bearing adult female nude mice.

## Data Availability

The datasets used and/or analysed during the current study are available from the corresponding author on reasonable request.

## Abbreviations

ART: Activity reference time
AUC: Area under the curve
CPM: Counts per minute
CRT: Chemoradiotherapy
DMEM: Dulbecco’s modified Eagle’s medium
EoI: End of Irradiation
EdU: 5-ethynyl-2’-deoxyuridine
FA: Fanconi anemia
FAAS: Flameless atomic absorption spectrometry
FCS: Fetal calf serum
HNSCC: Head and neck squamous cell carcinoma
HPV: Human papilloma virus
ICL: Interstrand cross-links
IP: Intraperitoneal
IV: Intravenous
MFI: Median fluorescence intensity
PBS: Phosphate buffered saline
