# Supplementary material for: Pharmacodynamics and biodistribution of [195mPt]cisplatin(CISSPECT®) in head and neck squamous cell carcinoma
Source: EJNMMI Res. 2024 Mar 1;14:22. doi: 10.1186/s13550-024-01082-w (PMC10904703; doi:10.1186/s13550-024-01082-w)
Supplement: Supplementary file 1 — Additional file 1. Supplementary materials and methods describing the production of Pt-195m radiolabeled cisplatin. [file 13550_2024_1082_MOESM1_ESM.docx]

**Materials and Methods**

1. [^195m^Pt]Cisplatin and cisplatin

Platinum-195m was produced by irradiation in the High Flux Reactor (HFR) in Petten, the Netherlands, in separate batches (Pt-0009, Pt-0011 and Pt-0016). After irradiation, [^195m^Pt]Cisplatin (CISSPECT^®^), or *cis*-[^195m^Pt ][Pt(NH_3_)_2_Cl_2_], was synthesized according to known procedures [1] as a 1 mg/ml solution in 0.9 % NaCl with a pH of 5 to 5.5. Part of the [^195m^Pt]Cisplatin solution, containing 0.065-0.067 mg Pt, was analyzed for radioactivity and radionuclide purity (^197^Pt, ^191^Pt ^192^Ir, ^194^Ir, ^198^Au, ^199^Au) using a high purity Germanium detector (HPGe) coupled to a multi-channel analyzer system. The energy window ranged from 50 to 1640 keV. Data were processed using NEMO software version 2.4.7 (NRG, van Dijken and Oudshoorn 2011). Ultraviolet-visible spectrophotometry (Perkin Elmer double-beam Lambda 365 UV-vis spectrophotometer) was used to identify Cisplatin by its characteristic absorbance at 301 and 365 nm. The absorbance at 301 nm was measured to determine the concentration of the [^195m^Pt]Cisplatin solution according to a calibration graph, which was plotted from the absorbance at 301 nm of solutions with concentrations of Cisplatin ranging from 0.2 to 1 mg/ml Cisplatin in 0.9% NaCl. The chemical and radiochemical purity were determined using High Performance Liquid Chromatography (Waters Acquity-arc HPLC system with a XBridge C8 column (3.5 μm) of 4.6 x 250 mm, UV-detection at 210 nm and detection of radioactivity with a Berthold Flowstar LB513 radiodetector) following procedures as described in the European Pharmacopeia (edition 7.0) with slight modifications. An aqueous solution of 1.08 g sodium octanesulfonate, 1.70 g tetrabutylammonium hydrogensulfate and 2.72 g potassium dihydrogenphosphate per liter, of which the pH was adjusted to pH 5.9 with 1 M sodium hydroxide, was filtered (0.2 μm Whatman RC58) and used as the mobile phase. A flow rate of 1 ml/min. was used and a run time of 10 min. European Pharmacopoeia reference standard Cisplatin CRS, Cisplatin impurity A CRS and Cisplatin impurity B CRS, were used to determine retention peaks of Cisplatin and of hydrolyzed Cisplatin (aquo complex), as well as of Transplatin (impurity A) and ammonium trichloroplatinate(‒) (impurity B).

Cisplatin (1 mg/ml concentrate for solution for infusion) was purchased from Accord (Accord Healthcare Ltd., United Kingdom).

**Results**

1. [^195m^Pt]Cisplatin

Specific activity per mg Platinum at End of Irradiation (EoI) was 81 MBq and 86 MBq ^195m^Pt/mg Platinum for Pt-0009 and Pt-0011 respectively, or 40 MBq and 42 MBq ^195m^Pt/mg [^195m^Pt]Cisplatin for Pt-0009 and Pt-0011 respectively (t½ = 4.02 d). At EoI, the radionuclide purity of Pt-0009 was 80.31% with ^197^Pt being the main impurity (19.68%, t½ = 19.9 hr) and 0.01% of ^191^Pt (t½ = 2.90 d). Radionuclide purity increased to 93.89% at the end of synthesis, Activity Reference Time (ART), 48 hrs after EoI due to decay of ^197^Pt (6.10%) and 0.01% of ^191^Pt present. Less than 0.01% of ^199^Au (t½ = 3.14 d) was present at ART. The radionuclides ^198^Au (t½ = 2.70 d), ^192^Ir (t½ = 73.8 d), ^194^Ir (t½ = 19.2 hrs) were below detection limits (not given). For Pt-0011, the radionuclide purity at EoI was 83.69% with ^197^Pt being the main impurity (16.29%) and 0.02% of ^191^Pt. Radionuclide purity increased to 93.61% at the end of synthesis (ART: 48 hrs after EoI) due to decay of ^197^Pt (6.10%) and 0.02% of ^191^Pt present. Less than 0.01% of ^198^Au and ^199^Au were present at ART. The radionuclides ^192^Ir and ^194^Ir were below detection limits (4.0E+01 Bq; 1.5E+03 Bq, respectively).

For batch Pt-0016, the specific activity per mg Platinum at EoI was 131 MBq ^195m^Pt/mg Platinum. Specific activity per mg [^195m^Pt]Cisplatin was 61 MBq ^195m^Pt/mg [^195m^Pt]Cisplatin at an ART of 48 hrs after EoI. At EOI, the radionuclide purity was 96.71% with ^197^Pt being the only impurity (3.29%, t½ = 19.9 hr). Radionuclide purity increased to 99.11% at the end of synthesis (ART: 48 hrs after EoI) due to decay of ^197^Pt. The radionuclides ^191^Pt, ^198^Au, ^199^Au, ^192^Ir, ^194^Ir were below detection limits (5.00E+02; 6.80E+01; 2.40E+02; 4.20E+01; 2.30E+03, respectively).

. [^195m^Pt]Cisplatin was dissolved in 0.9% NaCl and diluted to a concentration of 0.99 mg cisplatin/ml.

The UV-vis spectrum of [^195m^Pt]Cisplatin showed the characteristic absorbance of Cisplatin at 301 and 365 nm, thereby confirming the *cis* geometry of the synthesized complex. In contrast, Transplatin shows maximum absorbance at 270 and 315 nm, and displays a minimum absorbance at 295 nm (data not shown).Figure 1: UV-vis spectrum of the 1.0 mg [^195m^Pt]Cisplatin/ml 0.9 % NaCl aqueous solution.


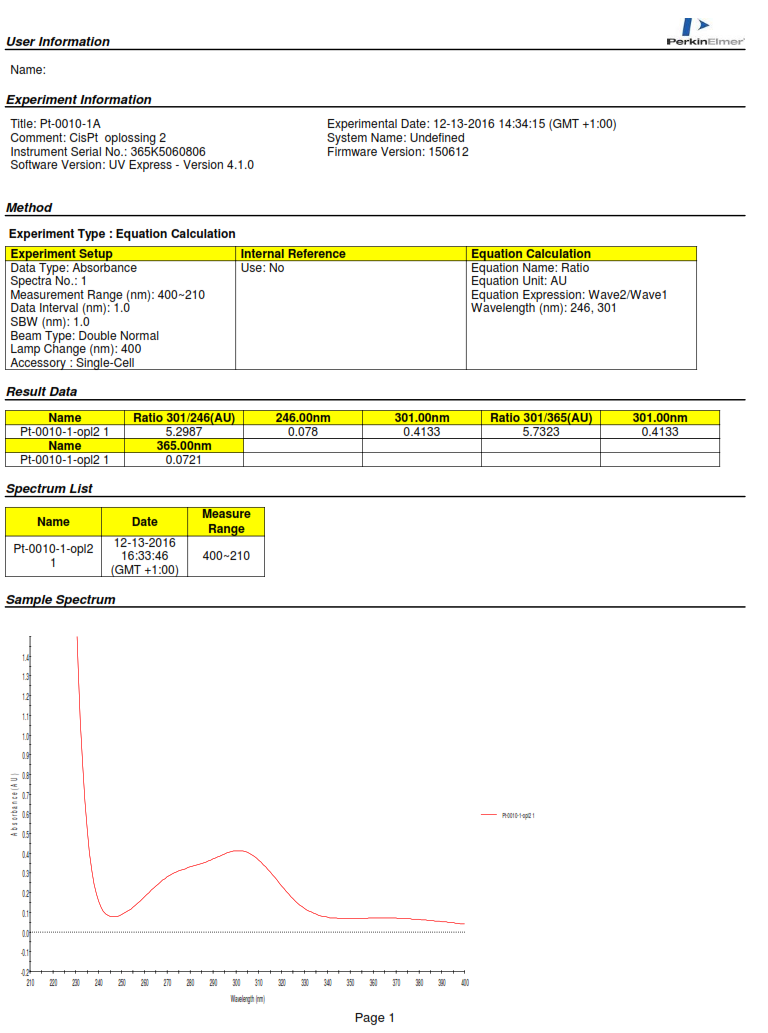


HPLC-UV runs of European Pharmacopoeia reference standards showed retention times for Cisplatin and hydrolyzed Cisplatin (aquo complex) of 4.1 and 5.3 min. (data not shown), respectively. Retention times of 2.8 and 3.0 minutes were shown for Transplatin (impurity A) and ammonium trichloroplatinate(‒) (impurity B) (data not shown), respectively. The HPLC-UV run of synthesized [^195m^Pt]Cisplatin shows retention peaks at 4.09 and 5.30 minutes only, with the peak at 2.42 min. being the injection peak. This indicates a chemical purity of > 99.9% if, in line with the European Pharmacoepoeia regulations for Cisplatin, the peak due to the aquo complex is disregarded. Retention peaks of [^195m^Pt]Cisplatin and its aquo complex in the HPLC radiochromatogram show a small time delay with respect to the UV chromatogram, due to the covered path length of the test sample from UV detection to radiodetection. The radiochromatogram confirms a radiochemical purity of > 99.9% as well.

Figure 2 HPLC-UV chromatogram of [^195m^Pt]Cisplatin of Pt-0009 and Pt-0011 (A) and Pt-0016 (B), HPLC radiochromatogram of [^195m^Pt]Cisplatin of Pt-0009 and Pt-0011 (C) and Pt-0016 (D).

A


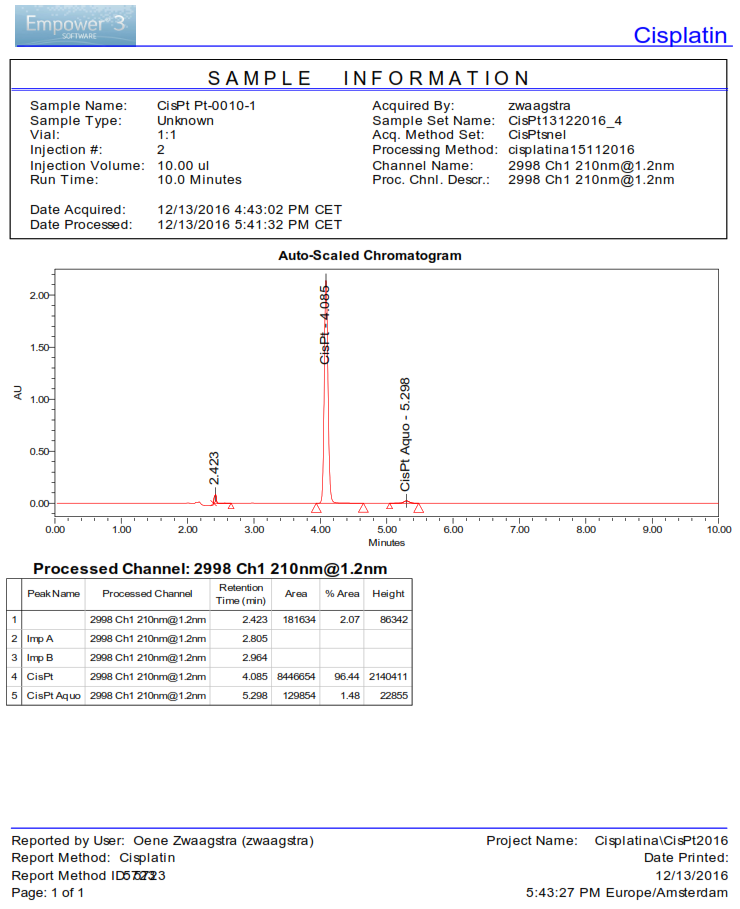


B


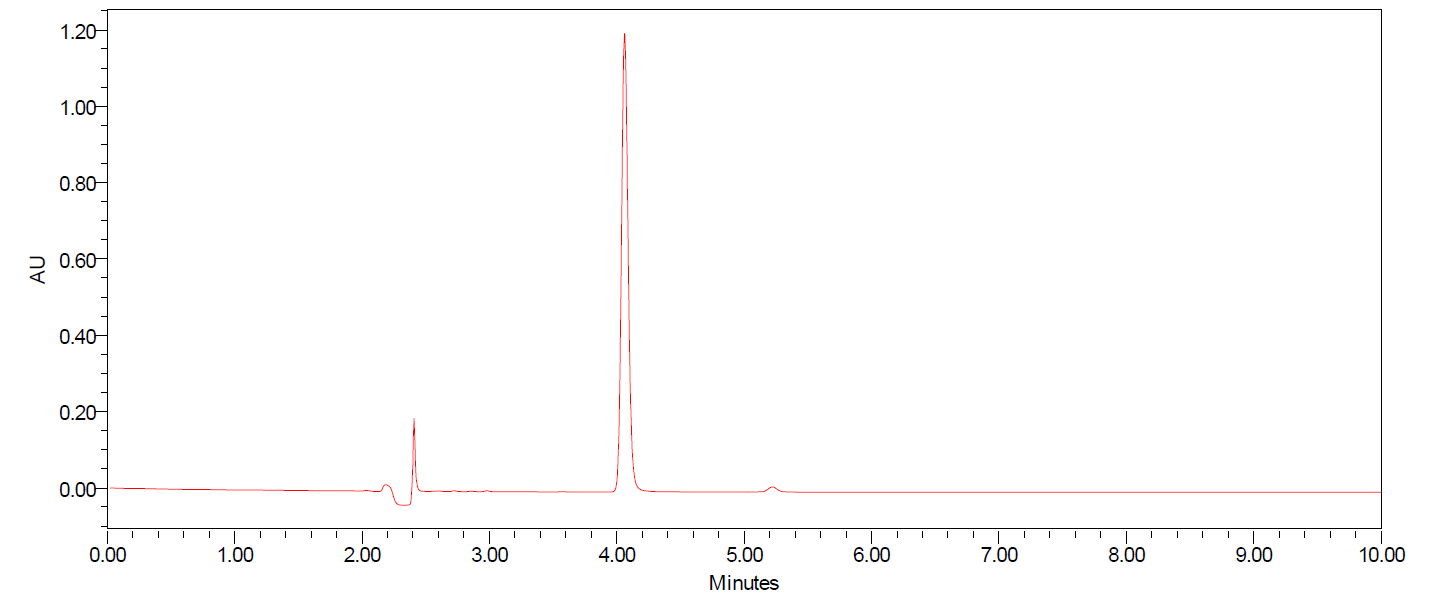


C


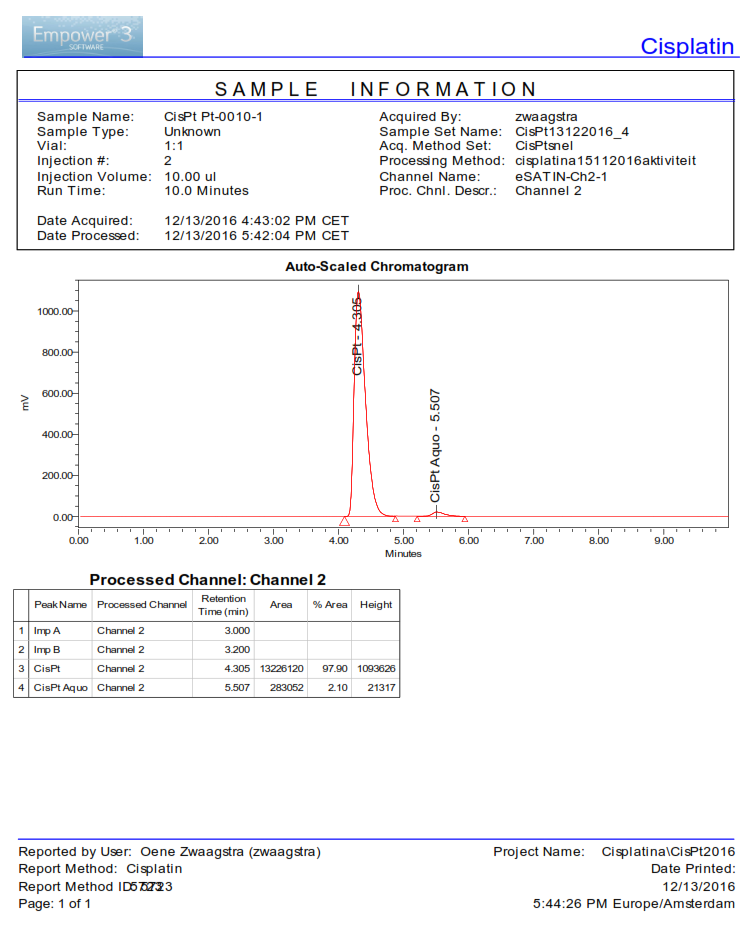


D


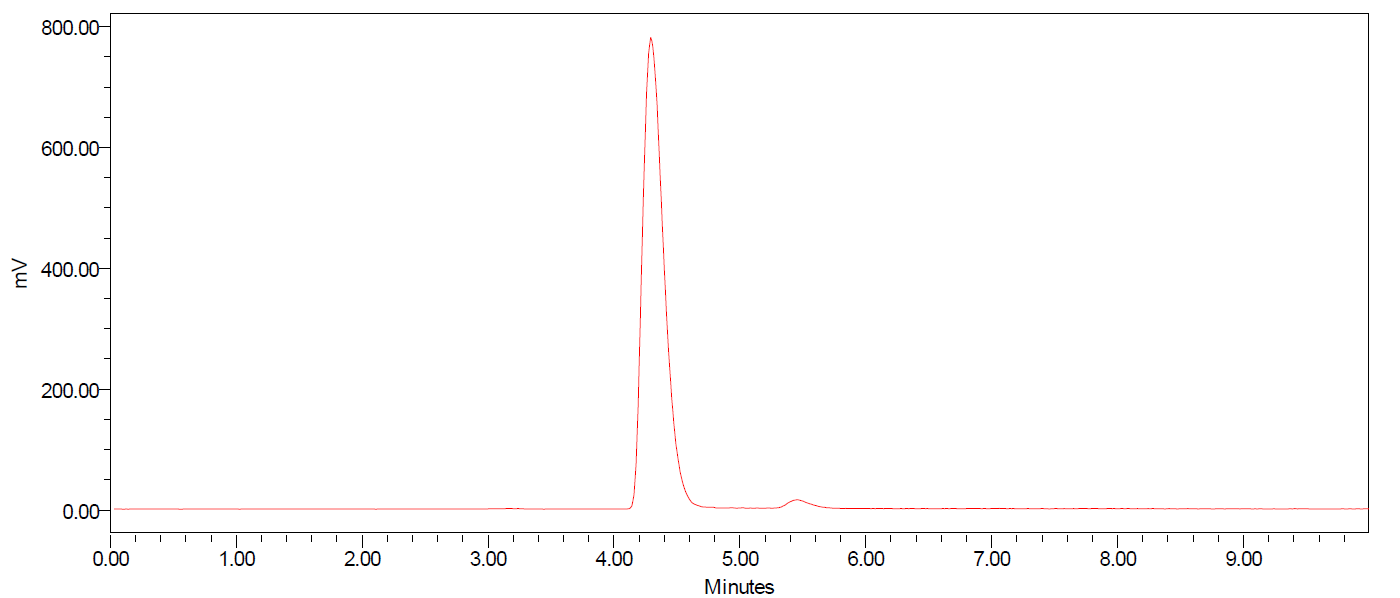


References

1. HOESCHELE, J.D., et al., Analysis and Refinement of the Microscale Synthesis of the 195mPt-labeied Antitumor Drug, cis-Dichlorodiammineplatinum(ll), cis-DDP*.* Radiochimica Acta, 1982; 31(1-2): 27-36. doi:10.1524/ract.1982.31.12.27.
