# Supplementary material for: Pharmacodynamics and biodistribution of [195mPt]cisplatin(CISSPECT®) in head and neck squamous cell carcinoma
Source: EJNMMI Res. 2024 Mar 1;14:22. doi: 10.1186/s13550-024-01082-w (PMC10904703; doi:10.1186/s13550-024-01082-w)
Supplement: Supplementary file 3 — Additional file 3. Supplementary table describing biodistribution of [195mPt] cisplatin in VU-SCC-1131 xenograft–bearing adult female nude mice. [file 13550_2024_1082_MOESM3_ESM.docx]

**Supplementary Figure 1A
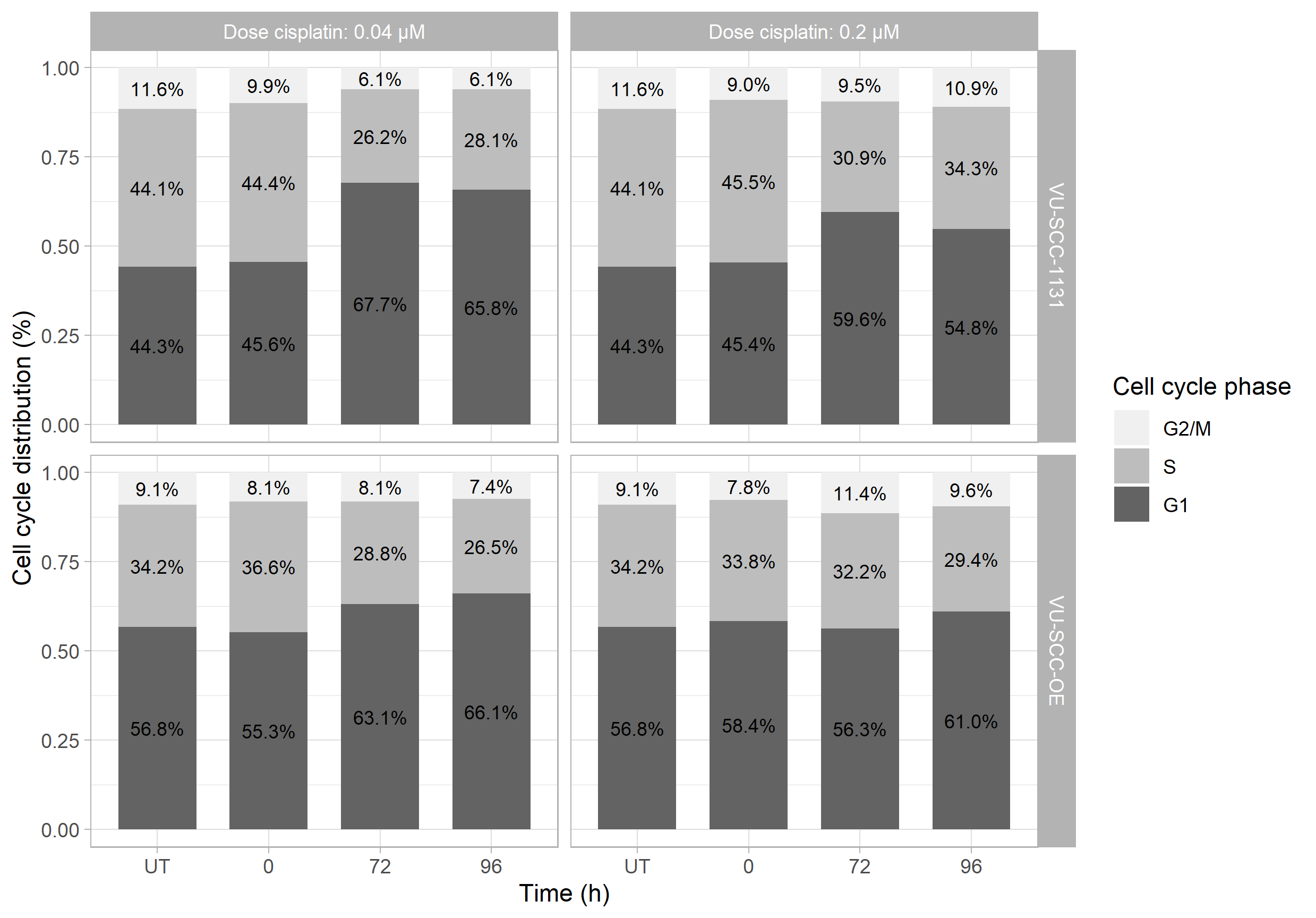
**

**Supplementary Figure 1B**

**
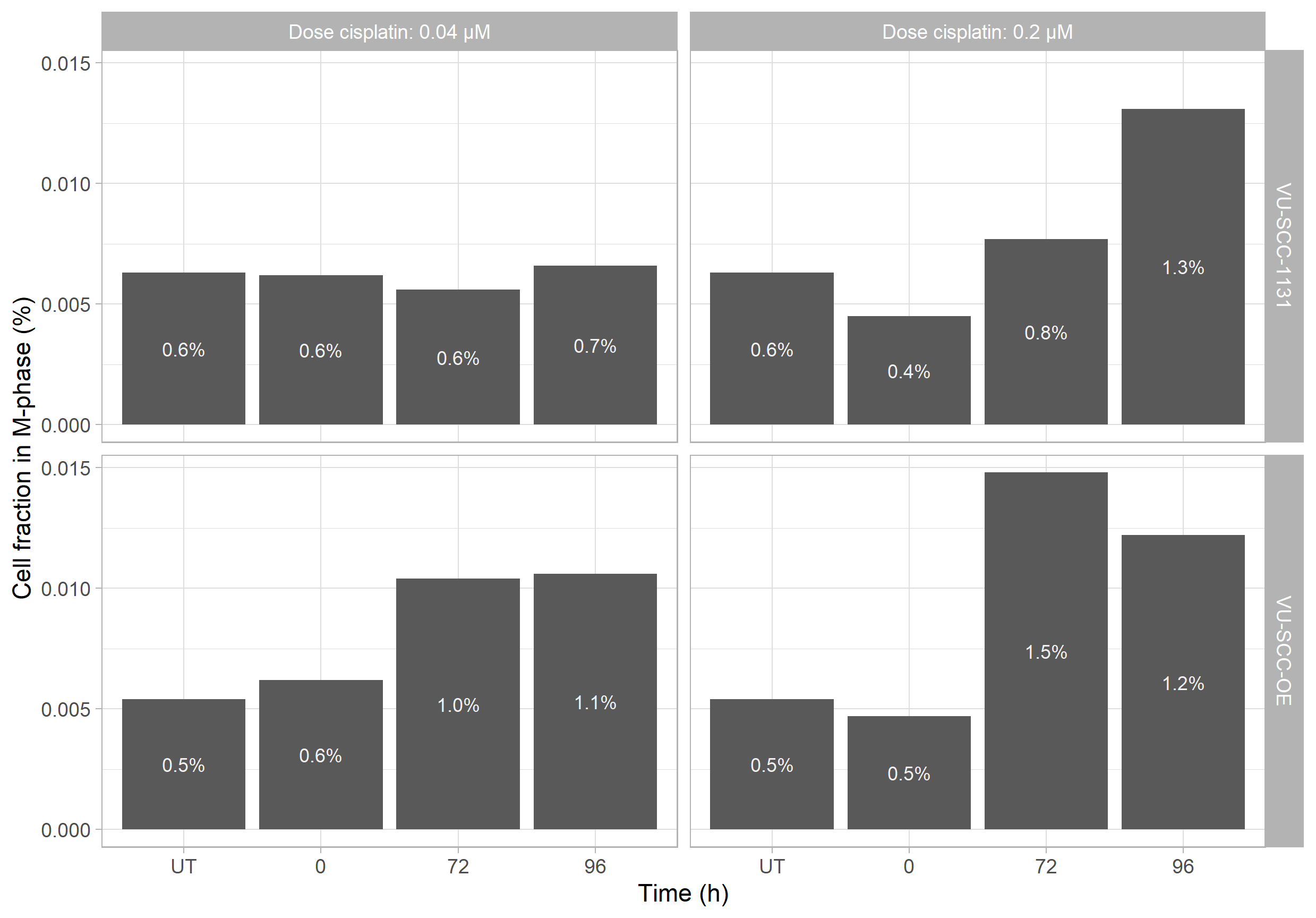
**

**Supplementary Figure 1:** Cell cycle distribution analysis after cisplatin exposure. Both cell lines were exposed for 4 hours with low dose (0.04 µM) cisplatin which correlates on our recovery experiment with the IC1.7 value of both VU-SCC-1131 and VU-SCC-OE, and high dose (0.2 µM) which correlates with the IC14.7 of VU-SCC-1131 and the IC7.0 of VU-SCC-OE. Cell cycle distribution was assessed directly after exposure and after 72h and 96h of repair time. A) Cell cycle distribution shows a higher proportion of cells in G1-phase after long repair time in VU-SCC-1131, VU-SCC-OE does not show alterations in cell cycle distribution. B) M-phase analysis shows an increased proportion of cells in M-phase after 72 and 96h of repair in VU-SCC-OE, this effect is only seen after high dose exposure in VU-SCC-1131

**Supplementary Figure 2
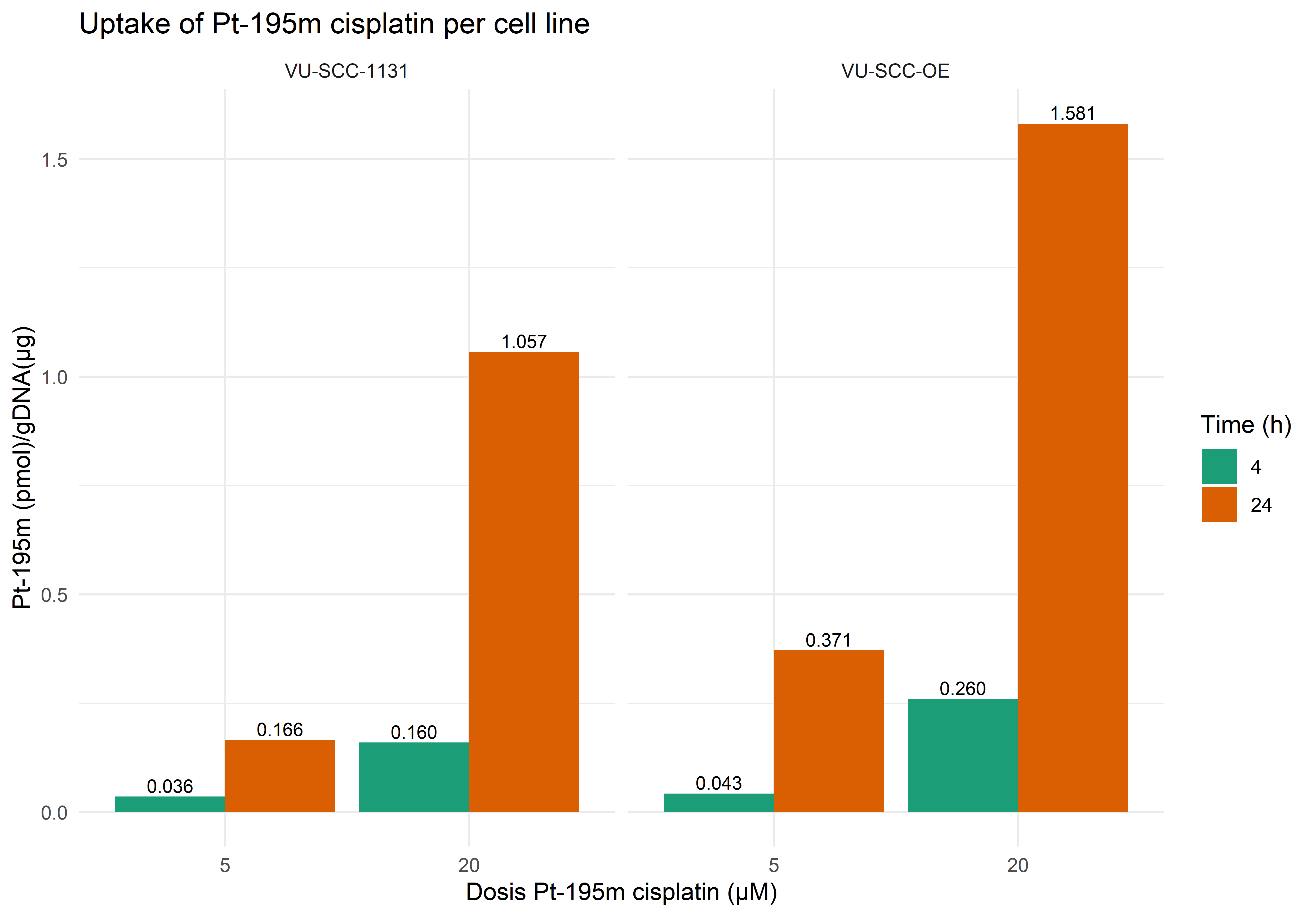
**

**Supplementary figure 2:** Shows the uptake and retention of Pt-195m cisplatin in the genomic DNA after 4 and 24 hours exposure of 5 µM of 20 µM Pt-195m cisplatin solution.

**Supplementary Figure 3**


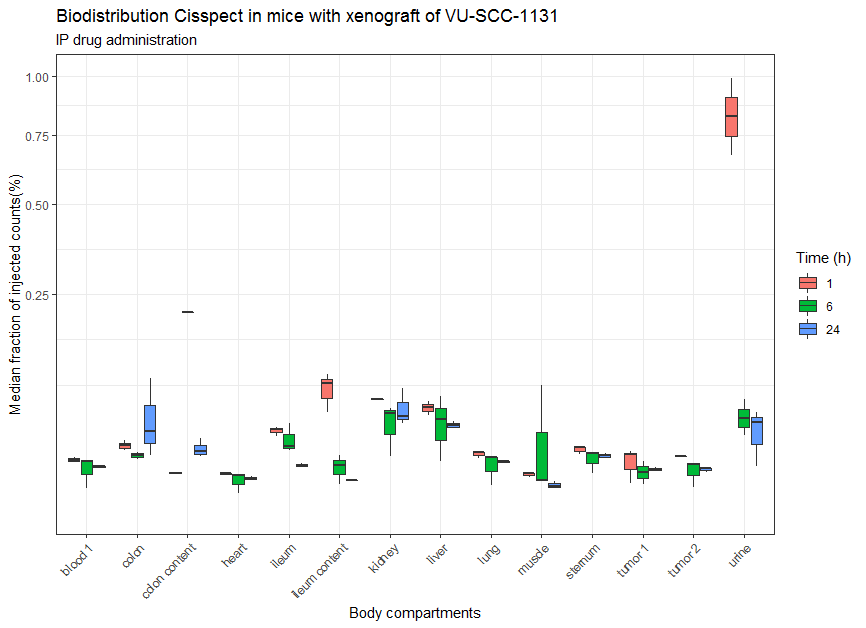


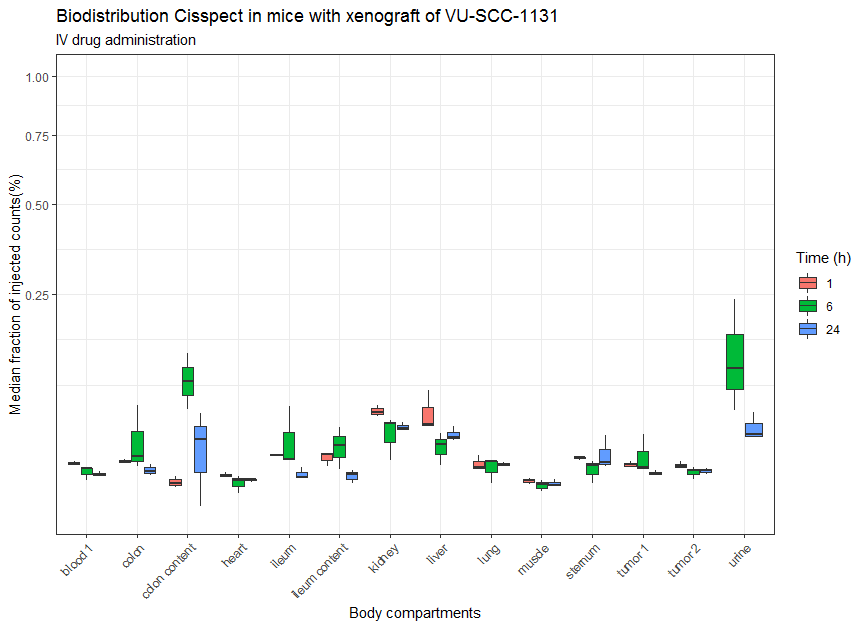


**Supplementary Figure 3:** Biodistribution of Pt-195m cisplatin in VU-SCC-1131 xenograft–bearing adult female nude mice (n = 3/time point) at 1,6 and 24 hours after intravenous injection. Differences between IV and IP administration. Radiotracer uptake in % injected dose/g was determined by γ-counting.

**Supplementary Figure 4**


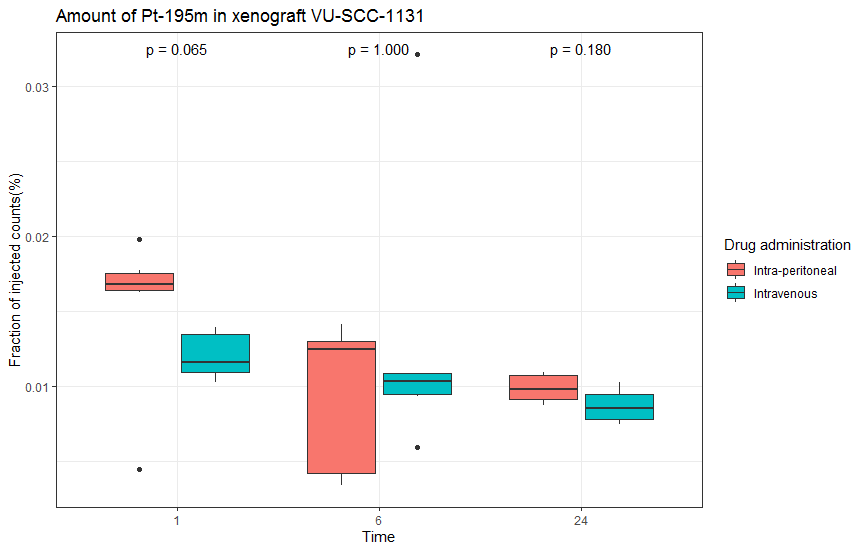


**Supplementary Figure 4:** Biodistribution of Pt-195m cisplatin in VU-SCC-1131 xenograft–bearing adult female nude mice (n = 3/time point) at 1,6 and 24 hours after intravenous injection. Differences between IV and IP administration in tumor uptake. Radiotracer uptake in % injected dose/g was determined by γ-counting.
